# Oncogenic Fusions Harboring *ETS* Genes: Exploring Novel Targetable Opportunities in Prostate Cancer

**DOI:** 10.3390/cancers17101657

**Published:** 2025-05-14

**Authors:** Federica De Cecco, Lidia Chellini, Veronica Riccioni, Maria Paola Paronetto

**Affiliations:** 1 Department of Movement, Human and Health Sciences, University of Rome Foro Italico, Piazza Lauro de Bosis, 6, 00135 Rome, Italy; dececcofederica91@gmail.com; 2 Fondazione Santa Lucia IRCCS, Via del Fosso di Fiorano, 64, 00143 Rome, Italy; chellinilidia@gmail.com (L.C.); veronicariccioni07011988@gmail.com (V.R.)

**Keywords:** prostate cancer, gene fusions, *ETS*, YK-4-279

## Abstract

Chromosomal rearrangements are implicated in the pathogenesis of several human malignancies, but, concurrently, they also represent targetable opportunities, as exemplified by imatinib (Gleevec), which targets the *BCR-ABL* gene fusion in myeloid leukemia. In prostate cancer, several chromosomal rearrangements have been identified, most of them involving *ETS* genes, which encode key transcription factors. In this review, we explore the discovery of 5′ partners that classify *ETS* gene fusions into distinct groups based on the prostate specificity and androgen responsiveness. Furthermore, we try to address the relationship between gene fusion status and patient outcomes and discuss the possibility of using prostate-specific targeting of *ETS* gene fusions in cancer detection, stratification, and treatment.

## Simple Summary

Recurrent chromosomal translocations are particularly common in human cancers, where they promote tumorigenesis by creating fusion genes from segments of two normal genes. These rearrangements can also disrupt tumor suppressor genes and lead to the amplification of oncogenes. This review examines the *ETS*-gene rearrangements in prostate cancer. A deeper understanding of the gene fusions in this disease could shed light on the clinical and molecular diversity associated with this widespread disease.

## 1. Introduction

Prostate cancer (PC) is the second-most common tumor diagnosed in men after lung cancer and the fifth leading cause of cancer death worldwide [1,2,3,4]. More than 1.2 million new cases are diagnosed every year, and global PC-related deaths exceed 350,000 annually [4]. Various genetic and demographic factors, such as age, family history, genetic predisposition, and race, play a role in the high incidence of PC. Epidemiological data indicate that the incidence of PC is also related to exposure to environmental chemical compounds. The sharp rise in industrial chemical production over the past 6–7 decades parallels the increasing rates of PC and other hormone-dependent cancers. With the widespread use of prostate-specific antigen (PSA) screening, nearly 90% of PCs can be diagnosed while still clinically localized [3]. The clinical behavior of localized PC varies significantly. Several risk stratification systems have been developed that integrate clinical and pathological factors like Gleason scores, PSA levels, and staging; however, these tools are still far from accurately predicting outcomes [1,2]. Although PSA is the primary biomarker used to detect PCa, it lacks specificity. Low PSA levels can still be present in aggressive PCa, while high levels can occur in benign cases. Additionally, prostate biopsies, the traditional detection method, are often uncomfortable and may yield false negatives if samples are taken from areas like the central or transitional zones where tumors may be absent. These limitations highlight the urgent need for better diagnostic and prognostic tools.

The natural history of PC onset and course is highly variable, making it a heterogeneous disease [5]. PCs that are detected early often advance slowly to a level where other aging-related causes/diseases pose a threat to life. Predicting the potential for early-stage cancers to progress is challenging. It is unknown whether genetic changes are linked to the onset and spread of PC despite extensive research being conducted on the molecular mechanisms underlying disease development [3]. At present, there is no infallible method to discriminate aggressive from indolent tumors. However, significant discoveries in the last century have dramatically changed the outlook for PC patients, including the hormone-dependent nature of PC [3] and the high therapeutic efficacy in targeting this key feature, specifically the high expression and frequent genetic amplification of the androgen receptor (AR). In addition, numerous studies for the genetic cause of this disease have revealed deletions of the *PTEN* gene and amplification of the *AR* in advanced disease [6].

Although AR plays a central role in PC, *AR* gene mutations are only observed in a minority of PCs and rarely at disease onset. This suggests that additional genetic lesions might contribute to prostate tumorigenesis. Advances in genome-wide analysis, microarrays, and high-throughput sequencing have allowed for the identification of key genes involved in the development and progression of PC, including aberrant fusion transcripts. Oncogenic chromosomal translocations involve the abnormal fusion of two genes, which can lead to deregulated or altered gene expression that are often, but not always, associated with the expression of chimeric proteins displaying transforming properties [6].

Translocations in *ETS* gene family members are the most common translocations implicated in PC development. It was initially reported that the 5′ untranslated region of *TMPRSS2* (21q22.2), which is prostate specific and androgen inducible, replaces the 5′ end of *ERG* (21q22.3) and *ETV1* (7p21.3) during PC development. Interestingly, the *TMPRSS2-ERG* fusion is often caused by a deletion [7], although a significant number of *TMPRSS2-ERG* fusions are formed through a translocation-like mechanism, whereas the *TMPRSS2-ETV1* fusion is always the consequence of a translocation [8]. *TMPRSS2* also fuses with other *ETS* family genes in a small percentage of PCs, such as *ETV4* (17q21) and *ETV5* (3q28). An interesting point of the *TMPRSS2-ETS* translocation is that, unlike IgH-related translocations, the product is not a chimeric protein; instead, overexpression of the oncogene occurs under the influence of a promoter element [9]. Other translocations identified in PC samples include fusions of *SLC45A3*, which encodes the solute carrier family 45 member 3, with *ETV5* (1q32.1)(3q28); *KLK2* (19q13.41) with *ETV4* (17q21); *C15orf21,* a prostate-specific androgen-repressed gene with *ETV1* (15q21.1)(7p21.3); and *SLC45A3* (1q32.1) with *ETV1* (7p21.3) [10]. Shan and colleagues found a translocation involving the *UNC5C* gene, located at the centromeric breakpoint on 4q22 [11]. This translocation directly causes the loss of the promoter region and the first exon of *UNC5C*, which encodes a putative tumor suppressor that is inactivated in various human cancers [12]. Notably, this translocation may contribute to PC development through the inactivation of tumor-suppressor genes rather than a gain of function by forming fusion genes [11].

This review explores the significant chromosomal translocations in PC and their involvement in the course and progression of the disease, and the therapeutic implications and opportunities.

## 2. The *ETS* Gene Family

The E26 transformation-specific (ETS) transcription factor family plays a crucial role in regulating various biological processes through its ability to bind specific DNA sequences, particularly in the promoter regions of target genes. With at least 28 genes categorized into five subfamilies [13], ETS transcription factors are involved in important pathways such as stem cell development, cell senescence, proliferation, migration, apoptosis, and tumorigenesis [13].

**Figure 1:**
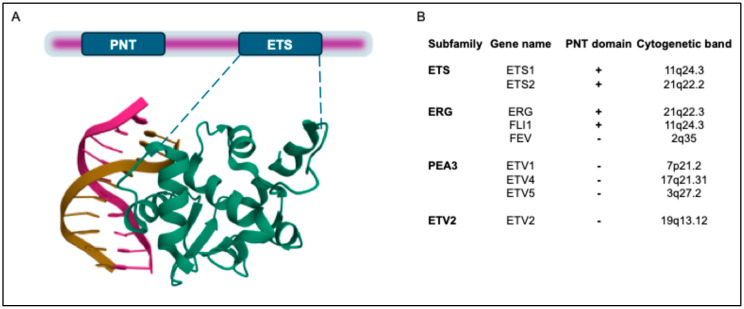
Domain structure of human ETS transcription factors (TFs) involved in oncogenic chromosomal rearrangements in PC. (**A**). In addition to the ETS domain, ETS1 and ETS2 also contain a Pointed (PNT) domain. In the lower part, the crystal structure of the ETS domain of ETS2 is shown, which was downloaded from the Protein Data Bank (PDB; https://www.rcsb.org/; https://doi.org/10.2210/pdb4BQA/pdb) [16]. (**B**). ETS transcription factors (TFs) have been classified into 12 subfamilies. Herein, only the proteins involved in PC translocations are shown. In addition to the ETS domain, a subset of the ETS family also possesses a PNT domain. Coordinates of cytogenetic bands of ETS genes are indicated.

All ETS transcription factors include a conserved ETS DNA-binding domain that consists of 80 amino acids and binds to the DNA core sequence 5′GGA(A/T)3′ (Figure 1A). The ETS domain is a variant of the winged helix–turn–helix motif [14], which is highly structurally conserved among the ETS family members, and contains three α-helices and four β-sheets (Figure 1A). Protein–DNA contacts occur through the third α-helix and in the ‘wing’ between β-strands 3 and 4, as shown in the crystal structure of the ETS domain of ETS2 [15].

ETS-domain proteins interact with various protein partners that either inhibit or enhance DNA binding. They also bind to coactivators and corepressors, which regulate their transcriptional activity. ETS-domain family members can be further sub-classified based on the presence of the Pointed domain, which was initially identified in the *Drosophila melanogaster* protein pointed (Pnt) [17]. The Pnt domain contains a bundle of four α-helices, with an additional N-terminal α-helix [18] that facilitates heterodimerization with protein partners and acts as a transcriptional repressor [17].

Among the genes involved in PC, *ERG* belongs to the “ERG subfamily” and contains a Pnt protein-interaction domain, while *ETV1*, *ETV4*, and *ETV5* belong to the “PEA3 subfamily”, so called because of their close homology with the epididymal transcription factor polyomavirus enhancer activator 3 (PEA3), who was the first member of this group to be found and whose human homolog is *ETV4* [19]. The ETS family members involved in PC chromosomal rearrangements are listed in Figure 1B.

ERG proteins interact with the Fos/Jun (AP1) complex to cause transcriptional activation and can form homodimer or heterodimers with other ERG splice variants and/or other ETS family proteins for reciprocal negative regulation [20]. Under normal physiological conditions, *ETV1*, *ETV4*, and *ETV5* display a role in morphogenesis; analysis of their mRNA levels revealed that these genes are widely expressed in several organs during embryonic development [19], motor coordination [21], and in fertility [22].

## 3. PC Chromosomal Translocations Involving ETS Family Members

Translocation of ETS genes were first reported in Ewing sarcoma (EWS-FLI1, EWS-ERG, and EWS-ETV1) and acute myeloid leukemia (TLS-ERG). The first time ETS genes were found to be involved in the development of human PC was the identification of the high expression of ERG in a large proportion of prostate tumor samples [8,23,24,25]. The most common chromosomal rearrangements involve the 5′ untranslated region of *TMPRSS2* (21q22) and the coding region of *ERG* (21q22), *ETV1* (7p21), or *ETV4* (17q21), leading to overexpression of the respective ETS family member [8,25]. The *TMPRSS2/ERG* rearrangement can be associated with aggressive tumor features [26], with incidences varying from 15% to 72% [27]. As a result of this translocation, a *TMPRSS2-ERG* fusion gene is created, with the expression of the ETS protein under the control of the promoter/enhancer of the *TMPRSS2* gene. Since *TMPRSS2* is androgen-inducible and is highly expressed in the prostate [28], its translocations lead to the androgen-dependent expression of ETS proteins in this organ in response to androgens, thus inducing ETS protein overexpression in PCs. *ERG* is the most commonly rearranged *ETS* gene in PCs (50%), primarily due to its proximity to the *TMPRSS2* gene on chromosome 21. The PEA3 family genes are localized on different chromosomes, resulting in less frequent translocations (*ETV1* translocations in 5–10% cases, and *ETV4* or *ETV5* translocations in ~1–2%) [29].

**Figure 2:**
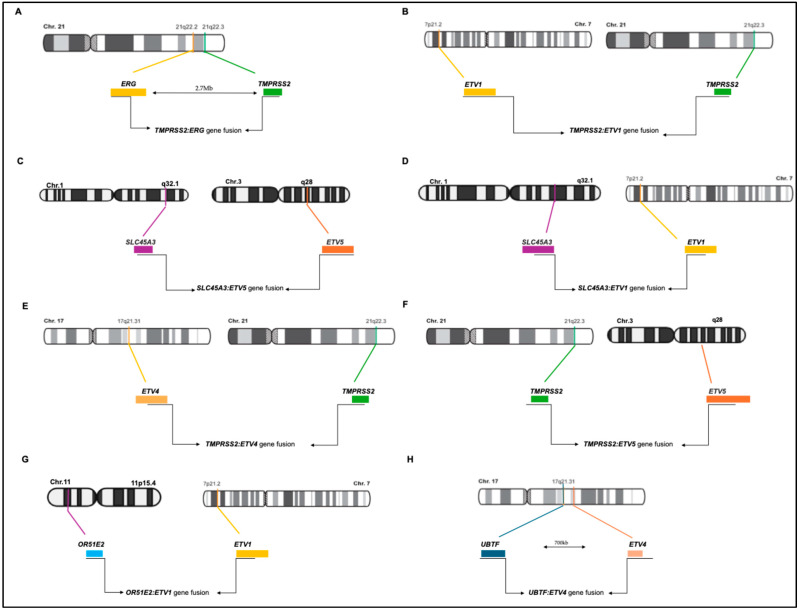
Schematic representations of *ETS* gene fusions in prostate cancer. (**A**). TMPRSS2:ERG gene fusion; (**B**). TMPRSS2:ETV1 gene fusion; (**C**). SLC45A3:ETV5 gene fusion; (**D**). SLC45A3:ETV1 gene fusion; (**E**). TMPRSS2:ETV4 gene fusion; (**F**). TMPRSS2:ETV5 gene fusion; (**G**). OR51E2:ETV1 gene fusion; (**H**) UBTF:ETV4 gene fusion.

*TMPRSS2* and *ERG* are about 3 Mbp apart in an identical orientation on chromosome 21 (Figure 2), and in about two-thirds of cases with *TMPRSS2-ERG* fusion, there is a complete or partial deletion of the middle sequence. Notably, more than 50% of these fusion events join the first intron of *TMPRSS2* with the third intron of *ERG* and lead to the most common *TMPRSS2-ERG* mRNA fusion transcript that juxtaposes exon 1 of *TMPRSS2* with exon 4 of *ERG* [9]. *TMPRSS2-ERG* fusion has been associated with a more aggressive phenotype in both clinically localized PC and metastatic PC [30]. Tomlins et al. reported the fusion of a 5′ sequence from *TMPRSS2* to a 3′ *ETV1* sequence; these genes are localized in two different chromosomes (Chr. 21 and Chr. 7, respectively). In the *TMPRSS2-ETV1* fusion event, exon 1 of *TMPRSS2* joins exon 4 of *ETV1*, leading to a rearrangement very similar to that of the *TMPRSS2-ERG* fusion gene [25].

In the *TMPRSS2-ETV4* fusion event, a short 8 Kb regulatory region located upstream of *TMPRSS2* and characterized by an androgen-regulated enhancer is juxtaposed to an intronic region immediately upstream of exon 3 of *ETV4*. While the upregulation of *ETV4* expression was shown in the RWPE, and PC-3 and DU145 cells, but not in LNCaP and VCaP cells, the *TMPRSS2-ETV4* fusion has not been reported in PC cell lines [31]. Remarkably, the downregulation of *ETV4* inhibits the proliferation and migration of PC cells and anchorage-independent growth in nude mice [31], whereas its overexpression stimulates proliferation, anchorage-independent growth, and migration of RWPE PC cells [31]. In the *TMPRSS2-ETV5* fusion event, exons 1–3 of *TMPRSS2* are fused with exon 2 of *ETV5*. *ETV1* and *ETV5* were also found to be fused with the androgen-induced gene solute carrier family 45 member 3 (*SLC45A3*). In these fusion events, exon 1 of *SLC45A3* is juxtaposed with exon 5 of *ETV1* and exon 8 of *ETV5* [32].

Later on, Barros-Silva and colleagues reported two new 5′ fusion partners of *ETS* genes (*OR51E2-ETV1* and *UBTF-ETV4*) [33]. The *OR51E2* and *ETV1* genes are mapped on chromosome bands 11p15 and 7p21, respectively, and share the same transcriptional orientation, indicating an origin from a chromosomal translocation. The *OR51E2-ETV1* transcript comprises the untranslated exon 1 of *OR51E2* with an additional 292 bp untranslated sequence downstream of exon 2 of *OR51E2*, which is fused to exon 8 of *ETV1*. The *OR51E2* gene (Olfactory Receptor, Family 51, Subfamily E, Member 2) encodes a member of the G-protein-coupled receptor family derived from a single coding exon. The *OR51E2-ETV1* fusion maintains the tissue specificity due to one of the two described promoters of *OR51E2* (in exon 1). The *UBTF-ETV4* fusion transcript comprises the untranslated exon 1 and 2 of *UBTF,* joined to exon 5 of *ETV4*. Both genes are in the same chromosomal band, 17q21, separated by about 700 kb.

Hermans and colleagues reported two other fusion genes involving *ETV4* in PC: Kallikrein 2 (*KLK2*)-*ETV4* and calcium-activated nucleotidase 1 (*CANT1*)-*ETV4*. *KLK2* is an androgen-regulated and prostate-specific gene, which maps to chromosome band 19q13, while *ETV4* maps to chromosome band 17q21. The *KLK2-ETV4* mRNA fragment comprises *KLK2* exon 1 linked to a new *ETV4* exon (exon 4a) followed by the *ETV4* exon 5 and 6 sequences. Because of the relative orientations of *KLK2* and *ETV4*, the *KLK2-ETV4* gene fusion cannot be accounted for by a single chromosomal translocation. The second *CANT1-ETV4* transcript contains one of the two described exons 1 of *CANT1* (exon 1a). This exon maps ≃4 Kbp downstream of the other first exon. Only one of the two alternative first exons of *CANT1* is present in the *CANT1-ETV4* fusion transcript. In contrast to most *CANT1* transcripts, this mRNA is preferentially expressed in the prostate. *CANT1* and *ETV4* map in the same orientation on 17q, at 35 Mbp [34].

DEAD (Asp-Glu-Ala-Asp) box polypeptide 5 (*DDX5*) has been identified as a new 5′ partner gene of *ETV4* by RLM-RACE in one PCa patient. Sequence analysis of *DDX5-ETV4* revealed that this fusion transcript comprises exons 1–3 of *DDX5*, fused in frame with exons 5–13 of *ETV4* [30]. Tomlins et al. described four 5′ fusion *ETV1* partners: *SLC45A3*; *HERV-K_22q11.23*, an endogenous retroviral element; *C15orf21*, a prostate-specific androgen-repressed gene; and *HNRPA2B1*, a strongly expressed housekeeping gene [25].

Although gene fusions can be considered a common feature in PC, their specific role in cancer development is still under investigation. *TMPRSS2* is a prostate-specific gene that is expressed in both normal and cancerous prostate tissue, whose expression is promoted by androgens in PC cells. Thus, the 5′ untranslated region of the *TMPRSS2* gene can drive the overexpression of the fused ETS gene in *TMPRSS2:ETS*-positive PCs in an androgen-dependent fashion [24,35]. During PC progression, ETS gene fusions likely act as genetic triggers, promoting the transition from a benign epithelium to prostatic intraepithelial neoplasia and leading to adenocarcinoma and metastatic disease [35]. In fact, *TMPRSS2:ERG* fusion is not expressed in benign prostate tissue, whereas it is found in localized PCs and in various metastatic stages. Furthermore, overexpression of *ETV1* in benign prostate cells increased cell invasion without affecting proliferation; however, transgenic mice overexpressing *ETV1* developed mouse PIN (mPIN), but not carcinoma [36,37]. These findings suggest that ETS gene fusions, while important, require additional genetic lesions (such as PTEN or NKX3-1 loss) to drive invasion and carcinoma development.

Initially, *TMPRSS2* was the only known 5′ partner in ETS gene fusions. However, as more cancers were studied, a lower percentage of *TMPRSS2:ETV1* fusions were found compared to *TMPRSS2:ERG* fusions, suggesting that other 5′ partners might be involved. Afterward, several new 5′ partners for *ETV1* fusions were identified, including *SLC45A3*, *HERV-K_22q11.3*, *C15ORF21*, and *HNRPA2B1*. All these partners are under the regulation of androgens, leading to distinct classes of ETS gene fusions [33].

Interestingly, transcription factors can contribute to genomic rearrangements by positioning chromosomal *loci* and recruiting enzymes that induce DNA double-strand breaks, thereby increasing the susceptibility of the transcribed regions to rearrangements. For example, the enzyme topoisomerase-2 beta (TOP-2b) is recruited to gene promoters in response to androgen and estrogen signaling, causing DNA breaks and aiding transcription. Androgen signaling itself has been shown to promote *TMPRSS2-ERG* fusion by recruiting DNA break-inducing enzymes and rearrangement breakpoints are more frequent near open chromatin and AR-binding sites in *TMPRSS2-ERG* fusions but less common in tumors lacking these fusions [38,39]. In line with these findings, inhibitors of repair enzymes like PARP1 and DNA-PK may reduce gene fusion susceptibility, while TOP-2b inhibitors, such as etoposide or doxorubicin, could promote gene fusions by enhancing DNA breaks. Thus, gene fusion vulnerabilities open the path to novel targetable opportunities in PC.

## 4. Strategies for Detecting Gene Fusions in PC

The detection of significant overexpression of *ETV1* or *ERG* genes in PC samples showing a consistent loss of the 5′ regions of the associated transcripts led to the identification of structural rearrangements involving chromosomal translocations. RNA ligase-mediated rapid amplification of cDNA ends (RLM-RACE), FISH techniques, and direct DNA sequencing have been instrumental to demonstrating that the 5′ end of *ETV1* or *ERG* are consistently replaced with the 5′ untranslated region of the prostate-specific gene *TMPRSS2* (21q22.2), generating different translocations (summarized in Table 1 and Figure 2). Remarkably, overexpression of either *ETV1* or *ERG* is mutually exclusive and associated with fusion transcripts, occurring in 10% of PCa cases for *ETV1* and 60% for ERG, thereby leading to the overexpression of their downstream targets [40,41].

The conventional methods for detecting chromosomal translocations are Fluorescence In Situ Hybridization (FISH), polymerase chain reaction (PCR), rapid amplification of cDNA ends (RACE), reverse-transcription PCR (RT-PCR), and real-time PCR (qPCR) [42]. FISH uses fluorescently labeled DNA probes targeting DNA sequences on chromosomes. FISH is fast, with a high resolution and it is often used to validate the results obtained from other methods. PCR methods are based on designing oligonucleotide primers on both sides of the breakpoint fusion region. This amplification allows for the identification of rearranged DNA fragments. RT-PCR is well established for identifying very small numbers of fusion transcripts. Both PCR and RT-PCR can be extremely sensitive and specific for the detection of chromosomal translocations. The application of qPCR to detect chromosomal translocation is more sensitive and rapid than RT-PCR. By using specific primers complementary to the breakpoint region, the fusion transcripts can be detected and quantified. All of these approaches, although highly sensitive, do not allow for the identification of novel chromosomal translocations. To this aim, next-generation sequencing (NGS) represents a powerful tool in the high-throughput approach for detecting translocations. Translocation detection is performed by sequencing the entire genome or specific regions of interest making it an uncommon approach in clinics due to its high cost and complexity.

**Table 1:** Chromosomal rearrangements identified in PC samples.

Translocation	Samples	Detection Technique(s)	Reference(s)
*TMPRSS2:ERG*	17/34		[43]
*TMPRSS2:ERG*	17/111	qPCR; FISH	[27]
*TMPRSS2:ERG;* *TMPRSS2:ETV4*	7/19 1/19	RT-PCR	[44]
*TMPRSS2:ERG*	44/63	RACE; RT-PCR; FISH	[45]
*TMPRSS2:ERG* *TMPRSS2:ETV1* *TMPRSS2:ETV4*	36/65 1/53 1/53	Interphase FISH	[27,46]
*TMPRSS2:ERG*	120/253	interphase FISH	[47]
*TMPRSS2:ERG*	11/26	RT-PCR; DNA sequencing	[48]
*TMPRSS2:ERG*	35/58 primary 3/7 lymph node metastases	FISH	[7]
*TMPRSS2:ERG*	35/86	RT-PCR; FISH; DNA sequencing	[49]
*TMPRSS2:ERG* *TMPRSS2:ETV1*	14/18 0	RT-PCR	[50]
*TMPRSS2:ERG* *TMPRSS2:ETV1*	35/82 1/82	RT-PCR; FISH	[51]
*TMPRSS2:ERG*	35/59	RT-qPCR	[26]
*TMPRSS2:ERG* *TMPRSS2:ETV1*	18/50 0	RT-PCR	[52]
*TMPRSS2:ERG*	6/15	RT-PCR; FISH	[53]
*OR51E2:ETV1*	1/14	5′ RACE	[33]
*CANT1:ETV4*	1/149	qPCR/FISH	[34]
*KLK2*:*ETV4*	1/149	qPCR/FISH	[34]
*DDX5:ETV4*	1/110	qPCR/FISH	[30]
*UBTF:ETV4*	1/14	5′ RACE	[33]
*SLC45A3:ETV1*	1/14	5′ RACE	[33]
*C15ORF21:ETV1*	1/14	5′ RACE	[33]
*HERVK17:ETV4*	1/14	5′ RACE	[33]
*HNRPA2B1:ETV1*	2/194	RT-PCR	[54]
*FLJ35294:ETV1*	1/110	qPCR/FISH	[30]

The development of new methods to detect genetic rearrangements has therefore improved the ability to identify new partners of the TMPRSS2 gene in PC. The improvement of NGS-based methods with fusion sequencing via terminator-assisted synthesis (FTAS-seq) allows for the enrichment of specific genes while profiling their 3′-terminal fusion partners. The detection of translocations based on NGS methods has several advantages compared to conventional methods, such as the detection of cryptic rearrangements and unknown partner genes, in addition to the possibility to analyze the presence of gene mutations in parallel [55]. In particular, using this semi-targeted RNA sequencing technique, 11 previously unknown TMPRSS2 fusion partners were identified (*Linc00114*, *PPP3CA*, *AMACR*, *CASZ1*, *SIM2*, *TTC18*, *FGFR2*, *OPTN*, *C1orf61*, *TBXAS1*, and *RERE*), with 21 different breakpoint sequences [56]. Moreover, with this method, various *TMPRSS2-ERG* isoforms in PC specimens were described [56]. In addition, next-generation mapping (NGM), a technology that is able to analyze megabase-length DNA sequences outside the detection range of single-base resolution NGS, allowed for the identification of large complex genome rearrangements, named chromoplexy, occurring coordinately and differently in ETS-positive and negative tumors, thus depicting the clonal progression of cancer genomes [57]. These novel approaches show promise as tools for biomarker discovery and could aid in the development of personalized cancer therapies.

Reliable detection of ETS gene fusions by immunohistochemistry (IHC) is hindered by antibody availability; while a reliable antibody exists for detecting ERG, other ETS gene fusions lack specific antibodies for cancer cells. This limitation highlights the need for more sensitive techniques, such as RNA in situ hybridization (RNA ISH). The advent of RNAscope technology has strongly improved detection sensitivity in comparison with traditional FISH techniques, while also minimizing background noise. The combination of IHC and RNA-ISH methods enables more comprehensive detections of ETS fusions. Kunju and colleagues validated the RNA-ISH method for the in situ detection of *ETV1*, *ETV4*, and *ETV5* in formalin-fixed paraffin-embedded PC samples [58]. By comparing RNA ISH/IHC with standard in situ techniques for detection of *ETS* gene rearrangements, they confirmed the reliability of the new method and demonstrated its utility in identifying rare PC subtypes with dual ETS gene rearrangements in different tumor foci of the same tumor [58].

The use of whole-mount radical prostatectomy has recently been instrumental to assessing the clonal heterogeneity of PC. This approach revealed mutually exclusive expression of various biomarkers across distinct tumor *foci* in multifocal tumors, with no single *focus* displaying the simultaneous expression of any of the other markers analyzed, indicating that the tumors likely arose through independent clonal evolution driven by distinct molecular aberrations [59]. With this technique, *TMPRSS2-ERG* fusion was confirmed as the most common genetic alteration in PC, with ERG overexpression in 56% of patients, followed by *SPINK1*, the second-most common biomarker, showing overexpression in around 10% of PC cases [59]. ERG expression was more frequently observed in younger Caucasian patients with low-grade, organ-confined tumors. In contrast, *SPINK1* expression was more frequently observed in African American patients, particularly in tumors with a higher volume (>20%) and anterior localization. Interestingly, 17% of cases showed *ERG* and *SPINK1* coexpression, either in different areas of the same tumor or in separate tumors within the same prostate gland, highlighting the heterogeneity of PC [59]. This multiclonal architecture can influence the Gleason grading, potentially affecting clinical decision-making by reclassifying patients from high-risk to intermediate-risk categories.

As mentioned above, biological differences across ethnicities were documented in PC, particularly in the frequency of ERG oncogenic activation. Notably, men of European descent have the highest rates of ERG alterations, while men of African or Asian descent have significantly lower rates [60]. Although these findings must be interpreted with caution due to variations in testing methods, sample types, and ethnic or geographical classifications, addressing these issues is crucial for realizing precision medicine for all PC patients [60]. These interesting observations highlight socioeconomic status as a contributing factor in the observed disparities; nevertheless, these emerging findings on ethnic differences in ERG frequencies could offer an objective way to assess ethnicity-based cancer biology to improve clinical diagnoses and enable tailored therapies.

## 5. Relevance of ETS Fusions in PC Prognosis

ERG gene rearrangements have been studied in various clinical settings, but no clear consensus on their significance has emerged. Some studies suggested that ERG fusions are linked to favorable outcomes, while others found no association, or even a connection to poor prognosis and more aggressive cancer [7,23,26,27,46,52,53,61]. ERG rearrangements involving deletions between *TMPRSS2* and *ERG*, as well as duplications of the deleted allele, have been linked to aggressive disease [7,46,62]. Further studies support this finding, documenting that duplication of the rearranged allele correlates with worse outcomes, including increased metastasis. However, in a subsequent study on a cohort of 521 patients undergoing radical prostatectomy, ERG rearrangements alone were not associated with disease stage or recurrence but were linked to a lower grade of cancer [63]. Additionally, research into other genetic alterations, like those involving *ETV1* rearrangements, has failed to show strong associations with poor survival [64]. This variability suggests that factors other than the ERG status, such as PTEN deletions and/or the expression/deletion of other factors, may contribute in determining PC progression and prognosis. *PTEN* gene deletion plays a major role in the development of PC. Yoshimoto and colleagues, in a small set of cases, observed that cancers with both *ERG* rearrangements and *PTEN* deletions had the worst prognosis [65]. These findings were supported by two in vivo studies involving transgenic mouse models. Carver and colleagues reported that PC samples with the *TMPRSS2-ERG* rearrangement often exhibited loss of the tumor suppressor *PTEN* [36]. Consistently, transgenic overexpression of ERG in mouse prostate tissue accelerated the progression of high-grade prostatic intraepithelial neoplasia (HGPIN) to prostatic adenocarcinoma in a heterozygous *Pten* background. Therefore, ERG seems to play a specific role in PC progression, working together with PTEN haploinsufficiency to drive the transition from HGPIN to invasive adenocarcinoma [36]. In parallel, King and colleagues demonstrated that transgenic *TMPRSS2-ERG* mice develop prostatic intraepithelial neoplasia (PIN) only in the context of PI3-kinase pathway activation when PTEN is inhibited or genetically ablated. They also showed that TMPRSS2-ERG-positive human tumors often exhibit PTEN loss, suggesting cooperation of these factors in prostate tumorigenesis [37].

Thus, the prognostic significance of these translocations remains unclear, although specific variants may have more predictive value. A key open question is whether the molecular understanding of ETS translocations can help in developing a new molecular classification of PC that differentiates cancers with distinct clinical behaviors. In this context, ERG overexpression alone does not seem to trigger PC; in vitro studies have shown that overexpressing *ERG* or *ETV1* increases cell migration and invasion [25,34,66,67]. Conversely, knocking down these genes in PC cells slows invasion [24,25,67,68]. In genetically modified mice, *ERG* or *ETV1* overexpression leads to prostatic intraepithelial neoplasia (PIN) but not invasive cancer and crossbreeding with *Pten*-knockout mice results in PIN and micro-invasive cancer. Thus, *ERG* appears to cooperate with other oncogenes or tumor suppressor genes in PC development.

Gene expression studies have identified pathways linked to ERG overexpression, such as the WNT and transforming growth factor β (TGFβ) pathways, as well as genes like *CACNA1D, TDRD1, PLA2G7*, and *NCALD* [44,69,70,71]. These genes may not be direct ERG targets but could be indirectly regulated or could mark cells where *TMPRSS2-ERG* fusion occurred. *TDRD1* has been identified as a direct ERG target [71]. Remarkably, ERG overexpression interferes with the AR gene expression program, potentially promoting dedifferentiation through EZH2 [72].

The role of *ETV1* in PC is less understood. In vitro studies showed that the full-length protein is a strong transcriptional activator, whereas the truncated form lacking the N-terminal TAD domain seems less active [73,74]. Both variants increase migration and invasion, but only the full-length protein induces anchorage-independent growth [73]. However, clinical samples displaying *ETV1* overexpression are rare, thus limiting a comprehensive understanding of *ETV1*-regulated genes in PC.

Thus, although *ERG* and *ETV1* both belong to the ETS family and may share some binding sites, they likely affect PC development through different mechanisms. ERG inhibits AR-regulated gene expression, while ETV1 appears to stimulate it, as evidenced by their opposite effects on PSA expression [72,75]. By using knock-in mouse models and genome-wide analyses, Baena and colleagues explored the distinct roles of *ERG* and *ETV1* translocations in PC. While both transcription factors regulate a shared set of genes, including AR targets, they perform this transcriptional regulation in opposite ways. ETV1 upregulates AR target genes and genes involved in steroid biosynthesis and metabolism, pushing for other oncogenic events, like PTEN loss, which contribute to more aggressive PC in both mice and humans [76]. These reported findings highlight biologically distinct PC subtypes defined by different ETS transcription factor signatures. Clinical outcome analyses confirmed that ETV1-regulated pathways are strongly linked to the progression of invasive prostate adenocarcinoma [76]. Specifically, gene sets defined by ETV1, but not by ERG, are associated with higher Gleason scores and metastatic disease. Consistently, ERG expression has shown no correlation with poor outcomes, and no significant association with Gleason scores, disease recurrence, or clinical prognoses [77].

Overall, the molecular evidence suggests that ERG and ETV1 have both shared and distinct targets, and clinical samples with ETV1-positive and ERG-positive tumors cluster separately [71,78], indicating limited evidence for a common mechanism in PC.

## 6. Therapeutic Approaches Targeting ETS Fusions

In PC cells, the overexpression of ETS factors enhances cell invasion and leads to the development of prostatic intraepithelial neoplasia (PIN) in transgenic mouse models [24]. Conversely, reducing ETS factors in vitro decreases cell motility and invasiveness. In line with these observations, depletion of *ERG* and *ETV1* was shown to be sufficient to slow tumor growth in vivo [25]. Recent studies have shown that *TMPRSS2-ERG* expression is reactivated in castration-resistant prostate cancer [35]. From these reported findings, ETS proteins have emerged as promising targets for preventing or treating metastatic PC. Several small molecule inhibitors have been identified in the past decade through in vitro screens (e.g., YK-4-279, BRD32048, and ERGi-USU) [79,80,81] and computer-aided drug design (e.g., VPC-18005) [82]. Since many ETS factors have known 3D protein structures, they represent suitable targets for structure-based drug discovery. However, while the protein sequences of ETS factors are conserved, limiting the development of oligonucleotides that mimic ETS1-binding sites [83], the 3D structures of their DNA-binding pockets and the homodimerization domains differ, with unique polar and hydrophobic regions, as well as amino acid variations. This diversity allows for the development of specific small molecule inhibitors for each ETS factor, expanding the potential for anti-ETS therapies, as discussed below (Table 2).

### 6.1. The Small Molecule Inhibitor YK-4-279

A small molecule inhibitor targeting the interaction between the oncogenic fusion protein EWS-FLI1 and its transcriptional partner DHX9 was developed for Ewing sarcoma treatment [79,89]. The RNA/DNA helicase DHX9 was shown to interact with EWS-FLI1 and enhance its oncogenic properties [89,90,91]. The YK-4-279 molecule was shown to target the chimeric protein EWS-FLI1 in Ewing sarcoma and impairs EWS-FLI1 activity and slow down tumor growth in mouse xenograft models [79,92]. Thus, YK-4-279 treatment, as well as DHX9 downregulation, could represent a targetable opportunity for Ewing sarcoma [92,93,94], and the TK-216 derivative has recently concluded phase I/II trial as a monotherapy and in combination with vincristine in relapsed or refractory Ewing sarcoma patients [95].

Since FLI1, ERG, and ETV1 are Class I ETS factors, sharing over 60% identity and 80% homology in their amino acid sequences [96], the YK-4-279 molecule was also tested in PC cell lines with androgen-dependent ERG and ETV1 expression [84]. Remarkably, the small molecule YK-4-279 effectively inhibited ERG- and ETV1-driven transcriptional activity, leading to decreased cell motility and invasion [84] and reduced primary tumor growth and metastasis of fusion-positive PC xenografts [85].

Since the 1940s, androgen deprivation therapy (ADT) has been the primary treatment for metastatic hormone-sensitive prostate cancer [3]. However, its initial effectiveness is short-lived, and most men eventually develop castration-resistant prostate cancer (CRPC), which leads to rapid deterioration and death [3]. For patients with CRPC, docetaxel-based chemotherapy is often used to extend survival and maintain quality of life. Prolonged use of high-dose docetaxel can lead to toxicity and resistance, leaving few treatment options for those who progress after or during treatment. Recently, drugs such as abiraterone, cabazitaxel, and enzalutamide have been approved for CRPC patients who no longer respond to docetaxel, but their effectiveness remains limited [97], thus highlighting the critical need to enhance the overall effectiveness of treatments while minimizing side effects. The combination of low-dose docetaxel and YK-4-279 was shown to significantly inhibit growth, trigger apoptosis, and reduce the migration and invasion capabilities of human PC cells [98].

### 6.2. Inhibition of ERG-DNA Binding Through DB1255 and the VPC-18005 Antagonist

DB1255 is a di-(phenyl-thiophene-amidine) compound and a DNA-binding modulator that specifically targets the ERG/DNA complex. It was identified in a screen of molecules affecting ERG/DNA binding [86]. The ETS domain binds to DNA by direct recognition (through conserved arginine residues) of the core 5′-GGA(A/T)-3′ sequence within the major groove, along with interactions with the phosphate backbone in the minor groove, that flanks the 5′-GGA(A/T)-3′ sequence [99]. Inhibition of ERG’s binding to DNA can be achieved by targeting a sequence that partially covers the minimal ERG-binding site and blocking ERG/DNA binding.

The search for antagonists designed to sterically prevent ERG DNA binding through its ETS domain to impair its transcriptional activity led to identification of the VPC-18005 inhibitor. A structure-based virtual screening approach was applied to the ERG-ETS domain crystal structure, identifying a druggable surface pocket overlapping with the ERG–DNA interface and adjacent to the DNA recognition helix (α3). The predicted small molecule would competitively block DNA binding. The identified lead compound, VPC-18005, directly binds to the ERG ETS domain and was shown to reduce migration and invasion of PC cells expressing ERG. Remarkably, VPC-18005 also decreased metastasis in a zebrafish xenograft model [82]. These results provide evidence that small molecules targeting the ERG-ETS domain can suppress transcriptional activity and reverse the transformed characteristics of PC with abnormal ERG expression [82].

### 6.3. Degradation of ERG by WP1130

The development of the WP1130 inhibitor stems from the rationale of targeting the ERG oncogenic program through inducing its degradation. The ubiquitin-specific peptidase 9, X-linked (USP9X), which is a deubiquitinase enzyme, binds to ERG in PC cells expressing *TMPRSS2:ERG* and deubiquitinates ERG in vitro. Knockdown of USP9X led to increased levels of ubiquitinated ERG, which was accompanied by a reduction in ERG levels. Remarkably, treatment with the USP9X inhibitor WP1130 was sufficient to achieve ERG degradation both in vivo and in vitro, thus decreasing the expression of genes associated with ERG, resulting in the inhibition of ERG-positive tumor growth in mouse xenografts [87]. Interestingly, the USP9X inhibitor WP1130 was also able to induce the degradation of pre-B cell leukemia homeobox-1 (PBX1) protein, which promotes PCa cell proliferation and confers resistance to doxorubicin and cisplatin [100]. The combined treatment with multiple deubiquitinase inhibitors, including betulinic acid or WP1130 and enzalutamide, reduced AR protein stability and mRNA expression in PC cells, making it an attractive strategy for CRPC treatment [101].

### 6.4. Inhibition of ETV1 by BRD32048

By utilizing small-molecule microarray screens, Pop and colleagues identified compounds able to affect the biological function of ETV1. In particular, BRD32048, a 1,3,5-triazine small molecule, was the leading candidate for modulating ETV1 activity. The authors found that BRD32048 directly binds to ETV1, influencing both its transcriptional activity and the ETV1-driven invasion of cancer cells. In addition, BRD32048 also inhibited the p300-dependent acetylation of ETV1, which, in turn, caused its degradation [80].

### 6.5. Inhibition of ERG Expression by ERGi-USU Inhibitor

To identify inhibitor small molecule compounds that can decrease ERG protein levels, a collection of 2407 small molecules, including natural products and approved oncology drugs, was used in a primary screen with a Western assay platform. The screening identified the ERGi-USU inhibitor, which inhibited the cell growth of ERG-harboring PC cells, whereas non-ERG-expressing cell lines were not affected [81]. Importantly, ERGi-USU significantly inhibited the growth of ERG-positive tumor xenografts in nude mice [81]. From the parent ERGi-USU compound, the more potent ERG inhibitor ERGi-USU-6 was developed by improving the synthesis procedure [102].

### 6.6. TNIK Kinase Inhibition by NCB-0846

By using mass spectrometry-based kinomic profiling, Lee and colleagues identified kinases that were differentially expressed and/or phosphorylated in DU145 cells engineered to express ERG. Among them, the authors identified Traf2 and Nck-interacting kinase (TNIK) as being markedly upregulated and phosphorylated upon ERG overexpression. This screen opened the path to the use of the TNIK inhibitor NCB-0846, which was able to reduce cell viability, colony formation, and anchorage-independent growth in ERG-positive PC cells [88].

### 6.7. Splice-Switching Oligonucleotides Targeting ERG Alternative Splicing

**Figure 3:**
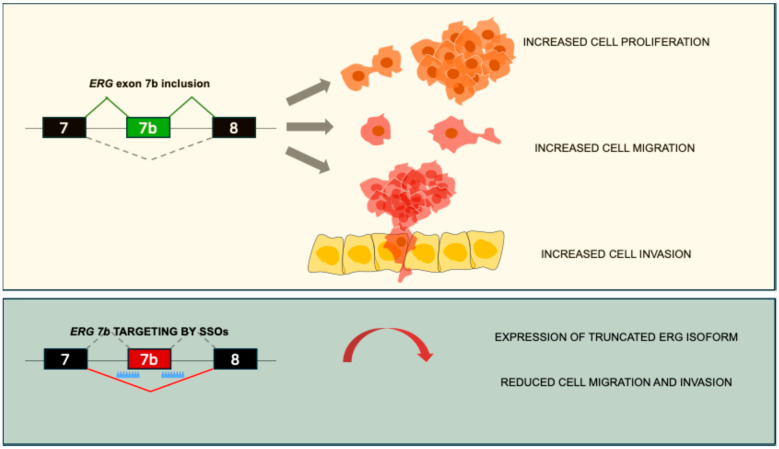
Splice-switching oligonucleotides targeting *ERG* alternative splicing represent a novel therapeutic approach for targeting ERG-positive PC. In the upper panel, *ERG* exon 7 inclusion leads to PC cell proliferation, migration, and invasion. In the lower panel, SSOs targeting exon 7b acceptor and donor sites lead to the exclusion of the alternative exon and recognition of a premature stop codon, leading to a truncated ERG protein [103,105].

With the aim of inhibiting ERG activity, Li and colleagues developed splice-switching oligonucleotides (SSOs) targeting ERG alternative splicing [103]. Alternative splicing amplifies the coding potential of the eukaryotic genome and increases the diversity of mRNA and protein products [104]. Regulation of splicing is essential for maintaining tissue homeostasis and is critically involved in development and various diseases, including cancer, suggesting that targeting this process could represent a promising strategy for therapeutic intervention [104]. Exon 7b of *ERG* is 72 bp long and adds, in frame, 24 amino acids to the transcriptional transactivation domain, whose inclusion is associated with advanced PC [105]. Reducing the inclusion of exon 7b through SSOs reduced cell proliferation and migration, and increased apoptosis, confirming the contribution of exon 7b to ERG’s oncogenic properties [105] (Figure 3). Treatment with a single dose of the SSO resulted in a marked drop in the ERG protein levels in both ERG-positive VCaP and MG63 cell lines and in mouse models. This led to decreased expression of key downstream target of ERG, including cyclin D1, c-Myc, and Wnt/β-catenin, thus reducing cell proliferation and migration, and promoting apoptosis [103]. These findings highlight SSOs as a potential therapeutic strategy for PC, like the successful Eteplirsen, which was developed for Duchenne Muscular Dystrophy treatment [106], and Nusinersen, developed for Spinal Muscular Atrophy treatment [107].

## 7. Conclusions

Genomic rearrangements represent a nonrandom and *locus*-specific feature of PC. Since its discovery in 2005, ERG overexpression through fusion with androgen-regulated genes has been linked to PC [8,23]. *TMPRSS2-ERG* fusion is present in about 50% of PCs and seems to be linked to disease progression. However, while its prognostic value remains unclear, ERG fusion shows promise as a diagnostic biomarker. Fusion genes can be detected via blood samples, enabling non-invasive diagnosis and monitoring. Moreover, gene-editing strategies targeting fusion breakpoints hold promise for future treatments. Overall, fusion gene profiling represents a valuable addition to PC management, improving prediction, guiding therapy, and offering mechanistic insights into disease progression.

Accurately predicting the clinical course of PC remains difficult, especially in identifying patients at risk of recurrence. While Gleason scores and serum PSA levels are commonly used prognostic tools, incorporating fusion gene profiling could significantly enhance the prediction accuracy for PC recurrence [108]. Machine-learning models combining fusion gene data with PSA and/or Gleason scores were able to achieve high levels of accuracy [108], proving their robustness and applicability in managing the molecular and phenotypic heterogeneity of PC.

Another issue to consider is that most genomic studies examine a single tumor focus, yet PC is often multifocal, with significant molecular heterogeneity across different regions of the prostate gland. This intra-tumoral heterogeneity should be considered in future genomic analyses, therapeutic development, and biomarker design.

The transrectal, ultrasound-guided 12-core systematic biopsy is currently the standard method for the initial diagnosis and grading of PC. Unlike diagnostic biopsies in other cancers, which usually target visible abnormalities, this method samples the prostate in a uniform but non-targeted manner. This approach often leads to missed cancer diagnoses and inaccurate grading at biopsy, with frequent changes in cancer classification after radical prostatectomy. Such diagnostic imprecision can result in overtreatment of low-grade disease or initial undertreatment of missed aggressive cancers. Magnetic resonance imaging (MRI)-targeted biopsies can better detect high-grade PC compared to standard biopsies, but it is still debated whether they should replace or supplement systematic biopsies [109]. Hence, future investigations should focus on cross-disciplinary approaches, integrating genomic profiling with artificial intelligence-driven digital pathology evaluations and high-throughput image analysis to facilitate precise, subtype-specific predictions of clinical outcomes and therapeutic responses. Harnessing interdisciplinary collaborations will deepen our understanding of the biological complexity of PC and expedite the discovery of robust predictive biomarkers to guide precision oncology strategies.

## Figures and Tables

**Table 2:** List of small molecule inhibitors targeting ETS transcription factors in PC cells.

ETS-Targeting Compound	Mechanism	Reference(s)
YK-4-279	Binds to ETS TFs and inhibits the interaction with DHX9	[84,85]
DB1255	Binds to a 5′-AA(G/N)T-3′ DNA and blocks ERG-DNA binding and transcription	[86]
WP1130	Inhibits USP9X activity, increasing ERG ubiquitination and degradation	[87]
BRD32048	Binds ETV1 directly, modulating both ETV1-mediated transcriptional activity and cancer cell invasion	[80]
VPC-18005	Binds ERG and inhibits its transcriptional activity	[82]
ERGi-USU	Specifically Inhibits ERG expression	[81]
NCB-0846	Inhibits the ERG dependent kinase TNIK	[88]
